# The effectiveness of the Ponseti method for treating clubfoot associated with arthrogryposis: up to 8 years follow-up

**DOI:** 10.1007/s11832-016-0712-1

**Published:** 2016-01-30

**Authors:** Hosam E. Matar, Peter Beirne, Neeraj Garg

**Affiliations:** Department of Trauma and Orthopaedics, Alder Hey’s Hospital, Eaton Road, Liverpool, L12 2AP UK

**Keywords:** Arthrogryposis, Clubfoot, Ponseti method

## Abstract

**Purpose:**

To evaluate the effectiveness of the Ponseti method in treating clubfoot associated with arthrogryposis.

**Methods:**

Retrospective consecutive review over a 10-year period in a tertiary centre of all patients with arthrogrypotic clubfoot treated with the Ponseti method. The primary outcome measure at final follow-up was the functional correction of the deformity.

**Results:**

There were ten children with 17 arthrogrypotic clubfeet, with an average follow-up of 5.8 years (range 3–8 years). The average age at presentation was 5 weeks (range 2–20 weeks). Deformities were severe, with an average Pirani score of 5.5 (range 3–6). Initial correction was achieved in all children with an average of 8 (range 4–10) Ponseti casts and a tendo-Achilles tenotomy (TAT) was performed in 94.1 %. Two-thirds of patients had a satisfactory outcome at final follow-up, with functional plantigrade, pain-free feet.

**Conclusions:**

The Ponseti method is an effective first-line treatment for arthrogrypotic clubfeet to achieve functional plantigrade feet. Children will often require more casts and have a higher risk of relapse.

## Introduction

Arthrogryposis includes a heterogeneous group of disorders characterised by multiple joint contractures, including clubfeet, flexed or extended knees, hip dislocations and upper extremity deformities [1–3]. Clubfoot in arthrogryposis tends to be severe, rigid, difficult to correct and has a high recurrence rate, making the goal of treatment “to convert a deformed, rigid foot into a plantigrade platform” [4]. Therefore, clubfoot is the most frequent indication for surgical treatment in children with arthrogrypotic syndromes [1]. Managing arthrogrypotic clubfoot has traditionally been through extensive soft tissue corrective releases and talectomy, with a high failure rate as well as complications [5–8].

The Ponseti method of manipulation and casting [9–12] is now considered the standard initial treatment for idiopathic clubfeet and is also thought to be useful in rigid, teratogenic clubfeet [13]. Few reports have been published on the results of the Ponseti method in the treatment of arthrogrypotic clubfoot [14–17]. In this study, we present our experience in treating arthrogrypotic clubfeet using the Ponseti method in our tertiary centre.

## Methods

This was a retrospective review of all patients with arthrogrypotic clubfeet treated at our institution between 2005 and 2012. In our tertiary hospital, we introduced the Ponseti method for treating clubfeet in 2002 and we established a dedicated weekly specialist Ponseti clinic in 2005, in which all clubfeet patients were seen by one of our senior authors (NKG) with an interest in paediatric foot conditions assisted by a team of trained plaster technicians and specialist physiotherapists [18, 19]. All patients underwent evaluation by a clinical geneticist and neurologist to confirm the diagnosis of arthrogryposis. On initial presentation, demographic data were collected, patients were assessed using the Pirani score [20, 21] by our senior author and the Ponseti protocol initiated. All patients were given written information about clubfoot and the Ponseti treatment. The standard Ponseti protocol was used with manipulation and high groin casting of the foot performed by the senior author. If necessary, tenotomy of the Achilles tendon was undertaken under general anaesthesia in the operating theatre. Following a successful initial correction, children were placed in a Mitchell boots and bar [10]. The external rotation in the boots and bar on the affected side was about 50–70°, depending upon the maximum external rotation achieved in the last plaster cast. This was worn full-time for 3 months, followed by wearing it at night and during nap time until 4 years of age (approximately for 14–16 h every day). Parents were given appointments to come back and see the orthotist to ensure compliance with the boots and bar. Children were followed up initially with 4-monthly clinical review for the first 2 years and then 6-monthly reviews. Given the lack of validated outcome measures for arthrogrypotic clubfoot, our primary outcome measure was the functional correction of the deformity, defined as achieving a plantigrade, pain-free foot. Secondary outcome measures included relapse and the need for surgical procedures.

## Results

There were ten children (five males and five females) with 17 arthrogrypotic clubfeet, with 7 (70 %) patients having bilateral deformities, with an average follow-up of 5.8 years (range 3–8 years) (Table 1). The average age at presentation was 5 weeks (range 2–20 weeks). Most deformities were severe, with an average Pirani score of 5.5 (range 3–6). Initial correction was achieved in all children with an average of 8 (range 4–10) Ponseti casts and a tendo-Achilles tenotomy (TAT) was performed in 16/17 feet (94.1 %).

**Table 1:** Demographics, treatment and outcomes of ten patients (17 feet) with arthrogrypotic clubfeet

Patient	Age (weeks)	Pirani score, R/L	No. of Ponseti casts	TAT	Recurrence	Follow-up (years)	Clinical outcome at final follow-up	Additional procedures	Ponseti method outcome
A	20	3/NA	4	No	–	3	Pain-free, plantigrade foot	AFO	Satisfactory
B	6	5/NA	8	Yes	Yes	6	Pain-free, plantigrade foot	2nd TAT	Satisfactory
C	3	5/5.5	10	Yes	–	6	Pain-free, plantigrade feet	AFO	Satisfactory
E	6	5/5	10	Yes	–	8	Pain-free, plantigrade feet	–	Satisfactory
F	2	NA/6	8	Yes	–	5	Pain-free, plantigrade foot	–	Satisfactory
G	3	6/6	9	Yes	–	6	Pain-free, plantigrade feet	AFO	Satisfactory
K	6	5.5/5.5	8	Yes	Yes, Bil whilst in hip spica	8	Pain-free, plantigrade feet	Four casts	Satisfactory
H	5	6/6	6	Yes	Multiple, poor compliance	8	Persistent deformity	Frame	Failure
I	3	6/6	7	Yes	Yes, Bil, 14 months	5	Persistent deformity	PMR	Failure
J	4	6/6	8	Yes	Yes, Bil, 24 months	3	Persistent deformity	PMR	Failure

*TAT* tendo-Achilles tenotomy; *AFO* ankle–foot orthosis; *Bil* bilateral; *PMR* posteromedial release

**Fig. 1:**
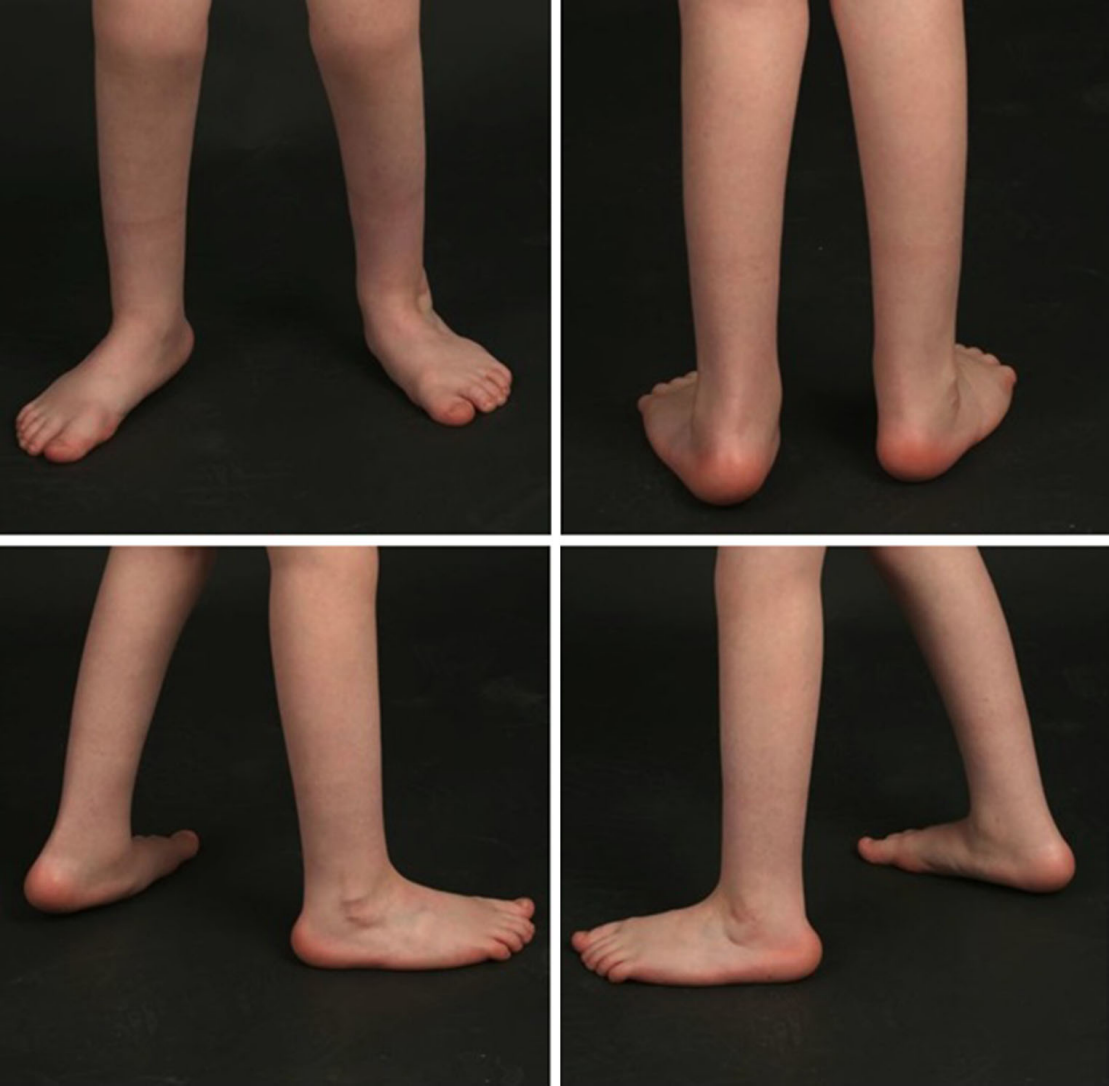
Clinical photographs of patient C at 6 years follow-up, with satisfactory outcome

Seven patients with 11/17 (64.7 %) arthrogrypotic clubfeet had a satisfactory outcome at final follow-up, with functional plantigrade, pain-free feet (Fig. 1). One patient had a recurrence that required a second TAT. Another patient had a relapse whilst in hip spica for bilateral dislocated hips and required a further four Ponseti casts. Three patients required additional use of an ankle–foot orthosis (AFO) to maintain the correction.

Three patients with bilateral severe deformities (6/17, 35.3 %) had failed Ponseti treatment, despite initial correction. All three patients had bilateral deformities scoring Pirani 6.0. One patient had multiple relapses, eventually requiring Ilizarov external fixator techniques [22], and two had persistent deformities requiring formal posteromedial soft tissue releases.

## Discussion

Following the remarkable success of the Ponseti method in treating idiopathic clubfeet [9], attempts were made to utilise this method in treating syndrome-associated clubfeet. In 2008, Morcuende et al. [16] published the first report of the Ponseti method in treating 16 patients with bilateral arthrogrypotic clubfeet with an average of 4.6 years follow-up. They reported satisfactory outcome in 11/16 (67.75 %) patients (Table 2).

**Table 2:** Summary of published studies on the use of the Ponseti method in treating clubfoot associated with arthrogryposis

	Boehm et al. [14]	Kowalczyk and Lejman [15]	van Bosse et al. [17]	Morcuende et al. [16]	Current study
No. of patients	12	5	10	16	10
No. of feet	24	10	19	32	17
Average follow-up, years	2	2.9	3	4.6	5
Satisfactory outcome	92 %	70 %	78.9	67.75 %	64.7 %

Only a few short-term follow-up studies have been published. In their short-term study (average 2 years follow-up), Boehm et al. [14] used the technique to successfully treat 12 patients with 24 clubfeet with distal arthrogryposis. Six feet had relapsed but were successfully treated by repeat casting, with an overall reported satisfactory outcome in 11 patients (92 %), with an average child age at final follow-up of 32.3 months [standard deviation (SD) 10.6]. In another short-term study, van Bosse et al. [17] reported satisfactory outcomes in 15/19 (78.9 %) arthrogrypotic clubfeet in ten patients using a modified Ponseti method with initial percutaneous Achilles tenotomy, followed by serial casting and a second tenotomy in 53 %, with an average follow-up of 3 years. Finally, Kowalczyk and Lejman [15] also reported on the short-term results in five patients with ten arthrogrypotic clubfeet treated with the Ponseti method, achieving satisfactory outcome in seven feet (70 %) (Table 2).

In the present study, we achieved satisfactory outcome, i.e. a plantigrade, braceable, pain-free foot, in 64.7 % of our children, with an average follow-up of 5.8 years (range 3–8 years). These results are similar to those published in the literature [14–17]. Although initial correction was achieved in all patients, maintaining the correction is rather challenging. Compliance with orthotics is paramount. This played a crucial role in our patients who relapsed and later required surgical release. It is worth noting, however, that these children often have complex needs and require a multi-disciplinary team approach to meet their rehabilitation needs.

To conclude, in our experience, the Ponseti method is an effective first-line treatment for arthrogrypotic clubfeet to achieve functional plantigrade feet, although children will often require a greater number of casts and have a higher risk of relapse.
